# Development of neural mechanisms of conflict and error processing during childhood: implications for self-regulation

**DOI:** 10.3389/fpsyg.2014.00326

**Published:** 2014-04-16

**Authors:** Purificación Checa, M. C. Castellanos, Alicia Abundis-Gutiérrez, M. Rosario Rueda

**Affiliations:** ^1^Department of Experimental Psychology, Faculty of Psychology, University of GranadaGranada, Spain; ^2^Developmental Cognitive Neuroscience Lab, Center for Research on Mind, Brain and Behavior, University of GranadaGranada, Spain

**Keywords:** executive attention, error processing, conflict resolution, self-regulation, development

## Abstract

Regulation of thoughts and behavior requires attention, particularly when there is conflict between alternative responses or when errors are to be prevented or corrected. Conflict monitoring and error processing are functions of the executive attention network, a neurocognitive system that greatly matures during childhood. In this study, we examined the development of brain mechanisms underlying conflict and error processing with event-related potentials (ERPs), and explored the relationship between brain function and individual differences in the ability to self-regulate behavior. Three groups of children aged 4–6, 7–9, and 10–13 years, and a group of adults performed a child-friendly version of the flanker task while ERPs were registered. Marked developmental changes were observed in both conflict processing and brain reactions to errors. After controlling by age, higher self-regulation skills are associated with smaller amplitude of the conflict effect but greater amplitude of the error-related negativity. Additionally, we found that electrophysiological measures of conflict and error monitoring predict individual differences in impulsivity and the capacity to delay gratification. These findings inform of brain mechanisms underlying the development of cognitive control and self-regulation.

## INTRODUCTION

Regulating behavior is effortful and requires attention particularly when relying on automatic well-learned actions is insufficient or impossible. Automatic behavior is not appropriate when alternative responses are available and the dominant more automatic response is not the desired one. In such situations errors are likely and detecting them also requires attention (Posner and DiGirolamo, 1998). Error-detection and conflict monitoring are mechanisms related to executive control and have been associated with activation of the executive attention network (EAN), a neural network involving the anterior cingulate cortex (ACC), the anterior insula, and other regions of the prefrontal cortex that are well connected with the basal ganglia and the autonomic nervous system (Posner et al., 2007). Thus, the EAN plays an important role in the regulation of thoughts and emotions (Rueda et al., 2011).

In the laboratory, conflict tasks such as the Flanker or Stroop-like tasks are used to measure executive control processes involving the EAN. Participants are slower and less accurate to respond to trials entailing conflict, as when distracting stimulation surrounds the target (flanker interference effect; Eriksen and Eriksen, 1974). Using this type of task with electrophysiological recordings allows studying the brain mechanisms related to executive control. Many studies have examined modulation of brain’s event-related potentials (ERPs) by conflict and have consistently shown that conflict modulates the amplitude of a negative deflection that appears around 200 to 450 ms after presentation of the target (N200, often also called N450; Liotti et al., 2000; Hanslmayr et al., 2008; Szucs and Soltész, 2012). This effect is distributed over mid frontal channels and has been related to activation originated in the ACC (van Veen and Carter, 2002).

Another ERP index associated with action regulation is the error-related negativity (ERN; Luu et al., 2003). The ERN is a negative deflection that appears around 100 ms after the commission of an error (Gehring et al., 1993). A widely accepted account of the ERN suggests that it reflects conflict at the response selection level, signaling a mismatch between the representation of the correct response and the one finally produced (Carter et al., 1999). The conflict monitoring account of ERN predicts activation of the EAN when detecting errors, and in fact both conflict monitoring and the ERN appear to have common cognitive mechanisms and shared neural basis (Yeung et al., 2004).

A second potential also modulated by the commission of an error is a positivity (P_e_) that arises around 200–300 ms after the response. This component is considered a later error-related signal, which reflects accumulated evidence that an error has been committed (Steinhauser and Yeung, 2010). The P_e_ has been associated with awareness of the commission of an error (Kaiser et al., 1997; O’Connell et al., 2007; Shalgi et al., 2009) and with the emotional significance of the error (Leuthold and Sommer, 1999; Ridderinkhof et al., 2009). The rostral part of ACC, a structure associated with self-referential thinking, is involved in the generation of P_e_ (Herrmann et al., 2004).

Over the course of development children become increasingly able to deal with conflict, showing a major development of this ability during the preschool years (Rueda et al., 2004a; Huizinga et al., 2006; Garon et al., 2008; Lee et al., 2013). Using conflict tasks adapted to children, it has been shown that young children (under age 7 years) show larger conflict effects compared to older children and adults (Rueda et al., 2005a). However, additional data with other tasks involving executive control indicate that this function shows a protracted development during childhood and depending on the demands of executive processes may extend to adolescence and early adulthood (Davidson et al., 2006; Waszak et al., 2010).

Electrophysiological studies have reported changes in brain activity during performance of conflict tasks with age. As adults, children show larger amplitude in trials involving conflict in ERP components around the expected latencies. However, compared to adults, conflict effects in children are larger in amplitude and duration, and have a more anterior distribution (Rueda et al., 2004b; Abundis-Guitiérrez et al., 2014). Moreover, the N450 effect decreases in amplitude with age, which some authors have interpreted as an index of improvement in efficiency of the EAN (Jonkman, 2006; Lamm et al., 2006; Espinet et al., 2012). Evidence from fMRI studies indicates that poorer performance on conflict task in children, compared to adults, relates to their ability to effectively recruit areas involved in cognitive control, such as the ventro-lateral prefrontal cortex (Bunge et al., 2002; Durston et al., 2002; Konrad et al., 2005).

Other studies have investigated the development of error processing during childhood. Errors can be caused by a premature execution of the response, and are often regarded as an instance of impulsive action (Botvinick et al., 2001; Pailing et al., 2002). This idea is supported by the fact that the reaction time (RT) in erroneous responses is usually faster than the RT in correct responses. Compared to adults, children show larger RT difference between correct and error responses (Davies et al., 2004a; Wiersema et al., 2007), indicating that children are more impulsive than adults, likely related to their greater difficulty in inhibiting inappropriate responses.

There is evidence that the ERN is present in children as young as 5 years of age when simple tasks are employed (Torpey et al., 2009). However, studies using more complex tasks have demonstrated that ERN is not clearly shown by children until late childhood (Davies et al., 2004a; Wiersema et al., 2007) or even until early adulthood (Ladouceur et al., 2007). Moreover, whereas the amplitude of the ERN has been positively correlated with age, the P_e_ appears to be more invariant across development than the ERN (Hajcak and Foti, 2008). Some studies have reported P_e_ effects of similar amplitude for children and adults (Davies et al., 2004a; Wiersema et al., 2007).

Over and above the existence of an ontogenetic developmental trajectory for the ability to regulate behavior, individuals show large differences in their self-regulatory capacities. Individual differences in regulation have been broadly studied in temperament research. Three broad dimensions characterize temperament during childhood and adolescence (Rothbart and Bates, 2006; Rothbart, 2007), namely: extraversion/surgency (E/S), negative affectivity (NA), and effortful control (EC). The first two dimensions describe individual differences in approaching and avoiding reactivity, respectively, whereas the third dimension describes individual differences in the ability to regulate emotions and actions in an internally guided or voluntary mode. EC is thus the temperament dimension most closely linked to the concept of self-regulation. Also, executive control mechanisms (i.e., conflict processing and error detection) have been conceptually and empirically linked to EC (Rueda, 2012). Many studies have shown an association between performance of conflict tasks and parent- or self- reported measures of EC (Gerardi-Caulton, 2000; Rothbart et al., 2003; Checa et al., 2008). Likewise, individual differences in conflict processing have been related to emotional regulation. It has been reported that children who obtain lower conflict scores show reduced tendency to frustration (Gerardi, 1997), less negative emotional reactions (Gonzalez et al., 2001), and better emotional regulation when facing challenging social situations such as receiving an undesired gift (Simonds et al., 2007). Moreover, low EC has been associated with disruptive behavior and poor sociability in school (Checa et al., 2008), presence of externalizing (Valiente et al., 2003; Olson et al., 2005; Eisenberg et al., 2009) and internalizing (Oldehinkel et al., 2004) behavior problems, and symptoms of depression (Verstraeten et al., 2009).

The current study had two main goals. First, we aimed at exploring the development of neural mechanisms related to conflict and error processing from early to late childhood. The second goal was to further examine the relationship between individual differences in functional efficiency of the EAN and behavioral and temperamental measures of self-regulation. For that purpose, children between 4 and 13 years of age and adults were asked to perform a child friendly flanker task while electrophysiological activity was recorded. The task was designed as to allow studying separately brain activation related to target and response processing. By using this procedure we intended to measure both the ERP related to conflict and error processing. Additionally, children’s self-regulatory skills were measured using a delay of gratification task and parent-reported temperament questionnaires. We expected a decrease in the size of the conflict-related potential as a function of age, primarily between preschoolers and older children. In addition, if larger amplitude on the conflict-related potential indexes poorer efficiency of the EAN, this effect should also be negatively related to behavioral self-regulation abilities. Finally, we expected to observe developmental changes in error processing from the preschool period to late childhood, and anticipated a positive relationship between efficiency of neural mechanisms related to error detection and children’s self-regulatory skills.

## MATERIALS AND METHODS

### PARTICIPANTS

A total of 20 adults (14 women; mean age = 23.6 years; SD = 2.6 years) and 47 children participated in the study. Children were divided into three groups: 4–6 year olds (*n* = 17, 10 girls; mean age = 5 years, SD = 1.04 years), 7–9 year olds (*n* = 15, 6 girls; mean age = 8.25 years; SD = 1 year), and 10–13 year olds (*n* = 15; 7 girls; mean age = 10.8 years, SD = 1.44 years). All participants were from an urban area of southern Spain, and had a similar social background. Information on mother’s educational level was collected for the sample of children according to a scale ranging from primary studies (1) to university degree (5). The average scores for children of the different age groups were 4.69 (SD = 0.11), 4.85 (SD = 0.10) and 4.87 (SD = 0.11), respectively, for 4–6, 7–9, and 10–13 year olds, which did not differ significantly from each other (*F* < 1). The parents of the children were contacted by phone and invited to participate in the study. They were part of a database of families who participated in prior studies and expressed their wiliness to participate in future studies. The adults were students of the University of Granada who signed up to participate in the study through the website of the department. The study protocol and recruitment procedures were approved by the Ethics Board of the University of Granada in accord with the Spanish Ministry of Science and Innovation norms for research involving humans. Participation was voluntary, and both the children’s caregivers and the adults gave written consent.

### PROCEDURE

Participants first completed the Flanker task while their brain activation was registered using a high-density (128-channels) electroencephalography (EEG) system. Fitting the sensor-net on, checking impedances, and completing the computer task took about 35 min, including brief breaks between blocks of trials. Once this task was completed, the sensor net was taken-off, and participants completed the self-report (adults) or parent-report (children’s caregivers) version of the temperament questionnaire, which took about 15 min. Finally, children completed a delay of gratification task. All participants performed the different tasks in the same order. At the end of the experimental session, a T-shirt of the lab and other small presents were offered to the children in appreciation for their collaboration. Adults received course credits in accordance to the norms of the Department of Experimental Psychology of the University of Granada.

### EXPERIMENTAL TASK

**FIGURE 1:**
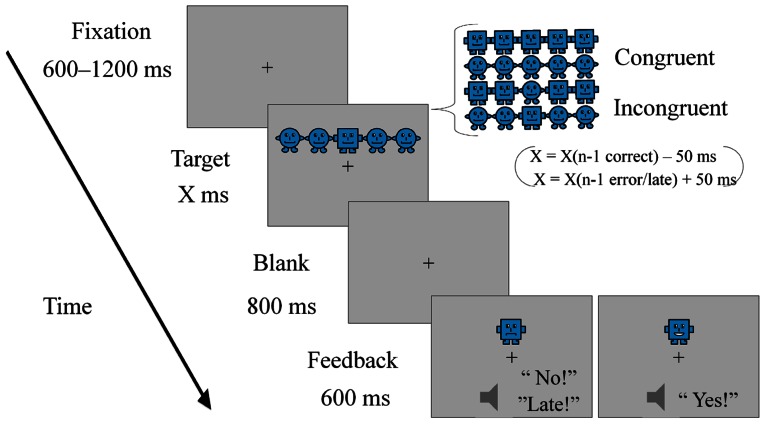
**Schematic representation of the experimental task used in the study**.

We designed a child-friendly flanker task using pictures of round and square robots as stimuli (see **Figure 1**). Each trial started with a fixation cross displayed at the center of the screen for a variable duration, randomly selected between 600 and 1200 ms. Subsequently, a cartoon picture of a row of five robots was presented either above or below the fixation cross. Participants were asked to focus on the robot in the middle and indicate whether it was round or square by pressing the corresponding button. The robot shape-to-response button mapping was counterbalanced across participants. Robots on the sides could be of the same (congruent) or different (incongruent) shape as that of the middle robot. Flanking robots were congruent in half of the trials, and the congruency condition was randomly selected for each trial. The response could be made during presentation of the target or up to 800 ms after it disappeared. The duration of the target was adjusted in each trial according to the participant’s performance in the previous trial. When an error was made, the response was omitted or given off time, the target duration was increased by 50 ms in the following trial. Alternatively, the target duration in trial n + 1 was decreased by 50 ms when the response in trial n was correct. Using this procedure, we intended to adjust the difficulty of the task across participants of different ages, as well as obtaining a significant number of errors in order to examine error potentials. Following the response, a 600 ms-lasting feedback was provided. The feedback consisted of a visual animation of the central figure plus an auditory word (“yes” for correct response, “no” for incorrect response, and “late” for omission or off-time responses). Participants completed 192 trials divided in eight blocks with small breaks between blocks.

### TEMPERAMENT QUESTIONNAIRES

The short form of the parent-report version of the Children’s Behavioral Questionnaire (CBQ; Putnam and Rothbart, 2006) was used for children between 4 and 8 years of age, whereas the Early Adolescence Temperament Questionnaire – Revised (EATQ-R; Ellis and Rothbart, 2001) was used for 9–13 years old children, and the Adult Temperament Questionnaire (ATQ; Rothbart et al., 2000) was used for adults. These questionnaires consist of a number of questions about people’s reactions in daily life situations that can be grouped into three main factors: EC, SU, and NA. The ATQ also includes a factor of orienting sensitivity (OS). The internal reliability for each factor in our sample was: Cronbach’s α = 0.60 for EC, α = 0.77 for SU, and α = 0.86 for NA in the CBQ; α = 0.85 for EC, α = 0.90 for AF, α = 0.34 for SU, and α = 0.63 for NA in the EATQ-R; and α = 0.53 for EC; α = 0.50 for SU; α = 0.65 for NA, and α = 0.82 for OS in the ATQ. Only the factors with α > 0.50 were included in subsequent analyses.

### DELAY OF GRATIFICATION TASK

We used a modified version of Thompson et al. (1997) Delay of Gratification task. We included six types of trial, which were created by crossing three types of reward (stickers, 5 cents of euro coins, and candies) and two types of choice: delay for oneself (DS) or delay for another person (DO). In the first condition (DS), children chose between obtaining: (a) a present for themselves immediately or (b) two presents for themselves at the end of the task. In the DO condition, children chose between obtaining: (a) a present for themselves immediately or (b) a present for themselves and a present for the experimenter at the end of the task. Each participant made 12 choices, 6 of each type. The dependent variable for this task was the percentage of delay choices.

### EEG RECORDING AND DATA PROCESSING

Electroencephalography was recorded using the 128-channel Geodesic Sensor Net (EGI Software: http://www.egi.com). Impedances for each channel were measured prior to recording and monitored during the EEG session. Channels with impedances exceeding 50 kΩ at recording were noted and discarded for further processing. The EEG signal was digitized at 250 Hz and 0.1–100 Hz band pass-filtered during the recording (time constant of 9 s). Recording in every channel was vertex referenced. After recording, data were filtered using a 0.3–12 Hz band pass filter. Continuous data were segmented in various ways in order to examine brain activation locked to different events: target and response. The epochs were 900 ms long (-200 to 700 ms) for target-locked ERPs and 1000 ms long (-600 to 400 ms) for response-locked ERPs. In both cases, we used the 200 ms prior to the event as baseline.

Segmented files were scanned for artifacts with the artifact detection tool provided by the EGI software Net Station. We used a threshold of 100 μV for eye blink or eye movements. Segments containing eye blinks or movements as well as segments with more than 25 bad channels were rejected. Data for each trial were also visually inspected to make sure the parameters of the artifact detection tool were appropriate for each participant. Individual ERPs data were included in the analyses as long as they had a minimum of 12 clean segments per experimental condition. The selection criterion was reached by 50 participants: 12 children in the 4–6 year group (7 girls, mean age = 5.1 years, SD = 0.9); 14 children in the 7–9 year group (6 girls, mean age = 8.1 years, SD = 0.93); 10 children in the 10–13 year group (3 girls, mean age = 11 years, SD = 1.1), and 14 adults (9 women, mean age = 26.5 years, SD = 5.3).

## RESULTS

### BEHAVIORAL RESULTS

**FIGURE 2:**
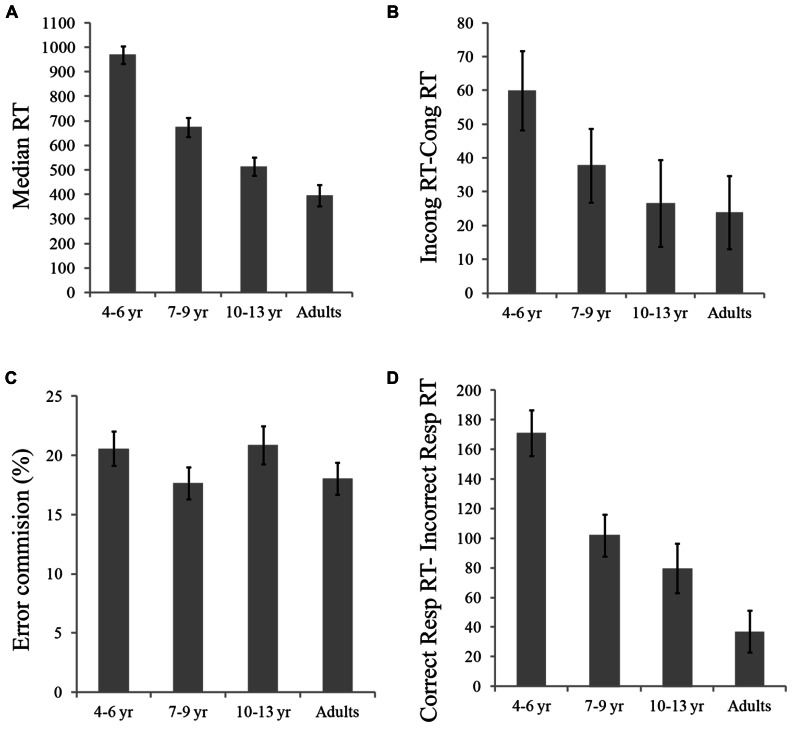
**(A)** Median RTs (ms) in each Age Group; **(B)** Flanker effect (RTs in incongruent – RTs in congruent trials) by age group in ms; **(C)** Percentage of commission of errors by age group; and **(D)** Impulsivity index (RTs in correct responses – RTs in incorrect responses) by age group, in ms.

Reaction time and accuracy data per age group in various conditions of the experimental task are presented in **Table 1**. Median RTs per experimental condition was used to measure speed of responses and percentage of errors (both errors of commission and omission) to measure accuracy. As shown in **Figure 2C**, the percentage of errors committed in the task was about 20% for all age groups, which provided sufficient error responses as to examine the brain reaction to errors and different types of feedback.

**Table 1:** Performance of the experimental task.

	% Errors				RT (ms)			
	OV-Com.	OV-Om.	Cong.	Incon.	Cong.	Incon.	Correct resp.	Errors resp.
	M (SD)	M (SD)	M (SD)	M (SD)	M (SD)	M (SD)	M (SD)	M (SD)
Adults	18 (5.5)	4.8 (1.1)	12.9 (6.0)	23.1 (7.2)	384 (31)	408 (31)	393 (29)	356 (29)
10–13 year	20.8 (2.9)	1.5 (1.2)	17.7 (4.3)	24 (4.8)	500 (59)	527 (57)	513 (58)	433 (70)
7–9 year	17.7 (5.3)	2.4 (2.5)	14.9 (4.7)	20.4 (6.9)	656 (131)	694 (134)	676 (137)	574 (123)
4–6 year	20.6 (5.4)	4.1 (3.3)	18.5 (6.0)	22.7 (7.0)	940 (205)	1000 (252)	965 (200)	794 (184)

*OV-Com., Overall commission errors; OV-Om., Overall Omission errors; M, Mean; SD, Standard Deviation; Cong., Congruent trials; Incon., Incongruent trials.*

Separate 4 (Age Group) × 2 (Flanker Type) ANOVAs with median RTs and percentage of errors as dependent measures were conducted. For RT, results revealed a significant main effect of Age Group, *F*(3,46) = 42.32, *p* < 0.001. Planned contrasts revealed that adults were faster than all children groups; [*F*(1,46) = 116, *p* < 0.001; *F*(1,46) = 30, *p* < 0.001 and *F*(1,46) = 4.5, *p* < 0.05 comparisons with 4–6, 7–9, and 10–13 years old, respectively]. Also, the 10–13 years olds were faster than the 7–9 year group, *F*(1,46) = 8.3, *p* < 0.01; and the 10–13 and 7–9 years old groups were faster than the 4–6 year group, *F*(1,46) = 62, *p* < 0.001 and *F*(1,46) = 31, *p* < 0.001, respectively (see **Figure 2A**). The main effect of Flanker Type was also significant, *F*(1,46) = 41, *p* < 0.001, indicating faster responses in congruent compared to incongruent trials. The Age Group × Flanker Type interaction was not significant, however, planned comparisons showed that the flanker interference effect (incongruent vs. congruent RT) was significantly larger for the 4–6 year group than for adults, *F*(1,46) = 5.12, *p* < 0.05, and marginally larger for the 4–6 year group compared to the 10–13 years old, *F*(1,46) = 3.7, *p* = 0.06 (see **Figure 2B**).

Using the percentage of errors as dependent variable, we found a significant main effect of flanker type, *F*(1,46) = 47.2; *p* < 0.001, indicating smaller percentage of errors in congruent compared to incongruent trials. Neither the main effect of Age Group, *p* > 0.5, nor the Age Group × Flanker Type interaction, *p* > 0.05, were significant on the accuracy analysis (see **Figure 2C**).

We also examined differences in RT for correct compared to incorrect responses across age groups. Overall, participants were faster when their responses were incorrect compared to correct, *F*(1,46) = 165, *p* < 0.001. The effect of Response Type interacted with age, *F*(3,46) = 14.16, *p* < 0.001. Planned contrasts indicated that the incorrect vs. correct difference in RT was smaller for adults compared to the 4–6 year, *F*(1,46) = 41.26, *p* < 0.001, 7–9 year, *F*(1,46) = 10.5, *p* < 0.01, and 10–13 year, *F*(1,46) = 3.7, *p* < 0.05, groups. Also, this difference was larger for the 4–6 year group than for the 7–9 and 10–13 year groups, *F*(1,46) = 10.9, *p* < 0.01 and *F*(1,46) = 16.1, *p* < 0.001, respectively, whereas there were no differences (*F* > 1) between children in the 7–9 and 10–13 year groups (see **Figure 2D**).

### DELAY OF GRATIFICATION

Percentages of delay choices obtained in the DoG task were entered in a 3 (Age Group) × 2 (Delay Type: self vs. other) ANOVA. Results revealed a significant main effect of Age Group, *F*(2,43) = 20.2, *p* < 0.001. Planned comparisons indicated that 7–9 years olds (65%) and 10–13 year olds (79.4%) did not differ on the percentage of delay choices. However, the percentage of delay choices was smaller for the 4–6 year group (27%) compared to the 7–9 year group, *F*(2,43) = 19.6, *p* < 0.001, and the 10–13 year group, *F*(1,43) = 37, *p* < 0.001. The main effect of Delay Type was also significant, *F*(1,43) = 5.9, *p* < 0.05, with larger percentage of delay choices for oneself (62.9%) than for someone else (51.8%). The Age Group × Delay Type interaction was not significant, *p* > 0.05.

### ERPs RESULTS

#### Target-locked ERPs

**FIGURE 3:**
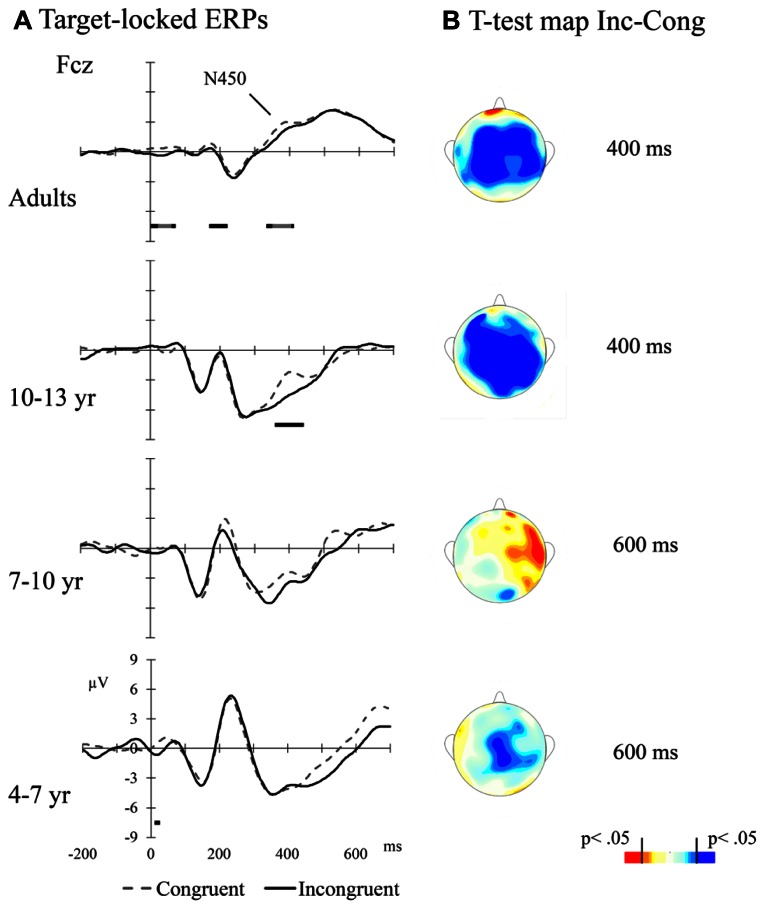
**(A)** Target-locked ERPs for adults and children at mid-frontal leads. The bars above the temporal scale show when the Error-Correct response *t*-test is significant (light gray: *p* < 0.01, black: *p* < 0.05, dark gray: *p* < 0.1); **(B)** Scalp distributions of incongruent – congruent contrasts at particular times after target presentation (*t*-test; 400 ms for adults and for 10–13 years old groups, and 600 ms for 7–10 year. old and 4–7 year old groups)

Averaged ERPs per Flanker Type condition and Age Group are presented in **Figure 3A**. **Figure 3B** illustrates the topographic distribution of incongruent minus congruent difference (congruency effect) at times of interest. The amplitude difference between congruent and incongruent trials appears to be largest between 350 and 450 ms for adults and older children, and some delayed for younger groups. In order to analyze the congruency effect in the different Age Groups, the mean amplitude per condition was calculated at different time windows: 350–450 ms post-target for adults and 10–13 year group, and 550–650 ms post-target for 7–10 and 4–7 year groups of children. Data from two lead positions over the midline, Cz, and Fcz, were included in this analysis. Thus, a 4 (Age Group) × 2 (Flanker Type) × 2 (electrode position: anterior-Fcz and posterior-Cz) ANOVA was run using the mean amplitude for the time windows specified above as dependent variable. The main effects of Age Group, *F*(3,46) = 4.04, *p* < 0.05, and Flanker Type, *F*(1,46) = 12.12, *p* < 0.01, were significant. The second indicating that the amplitude was more negative for incongruent compared to congruent trials. The Age Group × Flanker Type interaction was not significant (*p* > 0.1). The main effect of Electrode Position was also significant, *F*(1,46) = 126.95, *p* < 0.001, with larger amplitude at Fcz than at Cz. This effect was qualified by a significant Age Group × Electrode Position interaction, *F*(3,46) = 4.43, *p* < 0.001, showing that the Fcz vs. Cz amplitude difference was smaller in adults than in the 10–13 year, *F*(1,46) = 7.78, *p* < 0.001, and the 7–9 year, *F*(1,46) = 6.12, *p* < 0.05, groups. Also, it was smaller for the 4–6 year group compared to 10–13 year, *F*(1,46) = 7.12, *p* < 0.05, and 7–9 year, *F*(1,46) = 5.50, *p* < 0.05, groups. There was no difference between adults and 4–6 year group (*p* > 0.1), and between 10–13 year and 7–10 year groups (*p* > 0.1).

#### Response-locked ERPs

**FIGURE 4:**
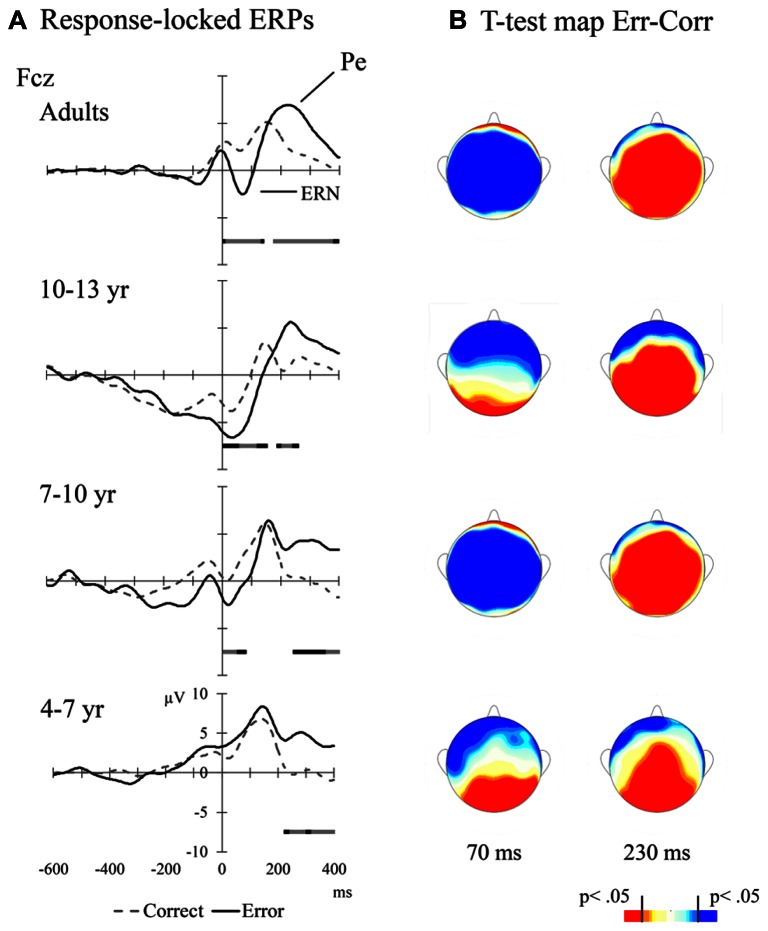
**(A)** Response-locked ERPs for adults and children at mid-frontal leads. The bars above the temporal scale show when the Error-Correct response *t*-test is significant (light gray: *p* < 0.01, black: *p* < 0.05, dark gray: *p* < 0.1); **(B)** Scalp distributions of the error vs. correct responses *t*-test values at particular times after the response (70 ms for ERN and 230 ms for P_e_ in all age groups)

**Table 2** shows mean amplitudes per condition and Age Group in the various ERP components of interest (i.e., N450, ERN, and Pe). Also, averaged ERPs for correct vs. error responses and the different age groups are presented in **Figure 4A**. **Figure 4B** illustrates the topographic distribution of the error minus correct responses difference at time points corresponding to the ERN and P_e_ peaks.

**Table 2:** Amplitude of ERPs components by channel, group, and conditions.

		N450			ERN		P_e_	
		Fcz	Cz		Fcz	Cz	Fcz	Cz
Group	Cond.	M (SD)	M (SD)	Cond.	M (SD)	M (SD)	M (SD)	M (SD)
Adults	Cong.	2.9 (2.4)	5.7 (3.1)	Co	1.6 (2.8)	4.7 (3.2)	5.4 (2.7)	7.5 (3.3)
	Incon.	2.3 (2.1)	2.8 (0.7)	Err	-2.8 (3.1)	0.4 (2.5)	7.7 (3.5)	8.8 (3.6)
10–13 year	Cong.	-2.8 (4.3)	2.9 (2.9)	Co	-4.1 (4.7)	3.5 (3.4)	4.4 (2.6)	8.7 (3.3)
	Incon.	-4.5 (4.2)	3.6 (1.1)	Err	-7.8 (6.0)	1.4 (5.1)	6.7 (5.2)	14.2 (3.8)
7–9 year	Cong.	1.4 (4.8)	6.2 (4.4)	Co	-0.2 (5.2)	4.3 (3.1)	6.8 (4.6)	7.5 (4.5)
	Incon.	0.4 (5.9)	5.1 (1.4)	Err	-3.7 (6.6)	2.4 (5.9)	7.6 (8.1)	11.9 (9.7)
4–6 year	Cong.	2.0 (4.3)	5.0 (3.1)	Co	1.3 (6.8)	2.2 (5.7)	7.2 (5.6)	5.2 (5.7)
	Incon.	0.1 (4.5)	3.8 (1.1)	Err	1.2 (5.4)	2.6 (4.9)	9.4 (6.7)	9.0 (6.4)

*The amplitude values are expressed in μVolts. M, Mean; SD, Standard Deviation; Cong., Congruent trials; Incon., Incongruent trials; Co., Correct responses; Err., Erroneous responses.*

A 4 (Age Group) × 2 (Response Type: correct vs. error) × 2 (Electrode Position: Fcz and Cz) ANOVA was run using a residualized ERN as dependent variable (see **Table 2**). This measure was calculated using linear regression to partial out the variability from the ERN amplitude due to the preceding positivity (see Santesso et al., 2005; Santesso and Segalowitz, 2008). The VD of the linear regression was the peak amplitude of the ERN at the time window from 0 to 100 ms post-response, and the VI was the peak amplitude of the preceding positivity at the -100 to 0 ms pre-response time window, and a residual score was saved. We found significant main effects of Response Type, *F*(1,46) = 29.61, *p* < 0.001, with larger negative amplitude for errors compared to correct responses; and Electrode Position, *F*(1,46) = 171.39, *p* < 0.001, with larger amplitude at Fcz than Cz. Both Response Type and Electrode Position interacted with Age Group, *F*(3,46) = 21.98, *p* < 0.001 and *F*(3,46) = 21.98, *p* < 0.001, respectively. The difference in amplitude between error and correct responses was significant in adults, *F*(1,46) = 27.04, *p* < 0.001, in 10–13 year group, *F*(1,46) = 8.41, *p* < 0.01, and 7–9 year children, *F*(1,46) = 10.31, *p* < 0.01, but not in 4–6 year children, *p >* 0.1. The Fcz amplitude was larger than Cz amplitude in adults, *F*(1,46) = 24.52, *p* < 0.001, 10–13 year, *F*(1,46) = 121.58, *p* < 0.001, and 7–9 year children, *F*(1,46) = 66.98, *p* < 0.001, but not in 4–6 year children, *p >* 0.1. The interaction Response Type × Electrode Position was significant, *F*(1,46) = 7.98, *p* < 0.01, because the error-correct response difference in amplitude was larger at Fcz than at Cz, *F*(1,46) = 7.21, *p* < 0.01.

Additionally, all age groups showed later larger positive amplitudes for error compared to correct responses (P_e_ effect; see **Table 2**). In order to analyze this effect, peak amplitudes per response type were calculated in a time window ranging from 130 to 270 ms post-response for each participant, and included in a 4 (Age Group) × 2 (Response Type) × 2 (Electrode Position: Fcz and Cz) ANOVA. The Response Type main effect was significant, *F*(1,46) = 24.77, *p* < 0.001, with larger positive amplitude for error than for correct responses. The Electrode Position main effect was significant, *F*(1,46) = 30.54, *p* < 0.001, with larger amplitude at Cz than Fcz. This effect was mediated by Age Group, *F*(1,46) = 12.21, *p* < 0.001, showing that the amplitude was larger at Cz than Fcz for adults, *F*(1,46) = 4.64, *p* = 0.036, 10–13 year *F*(1,46) = 45.12, *p* < 0.001, 7–9 year, *F*(1,46) = 11.30, *p* = 0.002, but not for 4–6 year children, *p* > 0.1. The Response Type × Electrode Position interaction was also significant, *F*(1,46) = 19.12, *p* < 0.001. This interaction was mediated by Age Group, *F*(1,46) = 6.56, *p* < 0.001. Planned comparisons indicated that the difference in P_e_ amplitude between errors and correct responses at Fcz was marginally significant in adults, *F*(1,46) = 3.94, *p* = 0.053, older children, *F*(1,46) = 3.06, *p* = 0.087, and younger children, *F*(1,46) = 3.25, *p* = 0.078 groups, but not in the medium children group, *p* > 0.1. At Cz, the P_e_ amplitude was larger for errors than correct responses in children groups [for older, *F*(1,46) = 16.85, *p* < 0.001; for medium, *F*(1,46) = 15.33, *p* < 0.001, for younger, *F*(1,46) = 9.53, *p* < 0.001] but not in adults, *p* > 0.1.

#### Correlations

Two scores of flanker interference (i.e., incongruent vs. congruent flankers) were obtained for each participant in both RT (FI_RT_) and percentage of errors (FI_ERR_). We also obtained an index of impulsivity (IM) by subtracting the median RTs for error responses from the median RTs for correct responses. Correlation between these scores and data on DoG and temperament showed that FI_ERR_ was positively correlated with impulsivity, *r* = 0.52, *p* < 0.05; *r* = 0.47, *p* < 0.05 after controlling by age. The percentage of delayed choices at the DoG task did not correlate with neither of flanker interference and impulsivity scores. The correlation between the FI_ERR_ and EC was significant in adults, *r* = -0.58, *p* < 0.05; and children younger than 9 years EC, *r* = -0.72, *p* < 0.001. FI_RT_ was also correlated with NA, *r* = 0.51, *p* < 0.05, and SU, *r* = 0.46, *p* < 0.05, for children younger than 9 years of age. The percentage of delayed choices at the DoG task did not correlate with any of the temperamental factors.

**FIGURE 5:**
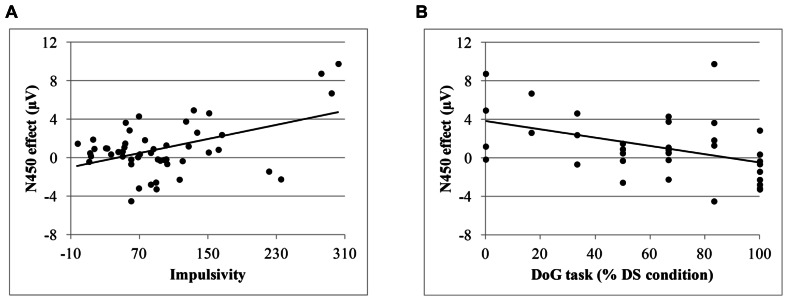
**(A)** Correlation between the N450 effect and the impulsivity score; **(B)** Correlation between the N450 effect and the percentage of delayed choices in the delay for oneself (DS) condition of the DoG task.

Additionally, we calculated ERP indexes of conflict (N450) and error processing (ERN and P_e_). The N450 index was obtained by subtracting the mean amplitude for congruent trials from the mean amplitude for incongruent trials at Fcz at the following time windows: 350–450 ms post-target for adults and 10–13 year children and 550–650 ms post-target for 7–9 year and 4–6 year children groups. The ERN index was calculated subtracting the residualized ERN amplitude for correct responses from the residualized ERN amplitude for incorrect responses at Fcz. The P_e_ index was calculated by subtracting the peak amplitude for correct responses from the peak amplitude of the error responses at time window at of 130–270 ms post-response at Cz. Pearson correlation between those ERP scores and indexes of task performance are presented in **Table 3**. We found a positive correlation between the N450 index and IM score, *r* = 0.47, *p* < 0.01; *r* = 0.49, *p* < 0.01 after controlling by age. Also, the N450 index was negatively related to the percentage of delayed choices in the DS condition of the DoG task, *r* = -0.36, *p* < 0.05 after controlling by age. These correlations are plotted in **Figure 5**. Additionally, the P_e_ index was correlated with IM, *r* = -0.27, *p* = 0.05, and with temperamental factor of SU, *r* = -0.32, *p* < 0.05; *r* = -0.27, *p* < 0.05, after controlling by age. Finally, significant correlation was also found between the ERN index and FI_RT_, *r* = -0.26, *p* < 0.05, as well as with the total of percentage of delay choices in the DoG task after controlling by age, *r* = 0.31, *p* = 0.05.

**Table 3:** Pearson correlations between electrophysiological indexes and indexes of performance of the experimental task.

		FI_RT_	FI_ERR_	IM	DOG
N450	Fcz	0.14 (0.11)	0.05 (-0.06)	0.47** (0.49**)	-0.34 (-0.36*)
ERN	Fcz	-0.26* (-0.21)	0.08 (-0.06)	-0.05 (0.02)	0.20 (0.31*)
P_e_	Cz	0.14 (0.07)	-0.17 (-0.10)	0.27* (0.15)	0.15 (0.08)

Data between parentheses are correlations controlled by age. Significance values: **p < 0.01; *p < 0.05.

## DISCUSSION

The aim of the current study was to investigate the neural mechanisms of executive attention and examine their relation to the development of self-regulation from early to late childhood. To asses executive attention we used a child-friendly flanker task designed to measure conflict resolution, as well as error and feedback processing. In this task, the duration of the target was adjusted in a trial-by-trial basis for each participant in order to ensure an equivalent level of task difficulty for participants of different ages.

### DEVELOPMENT OF CONFLICT AND ERROR PROCESSING

Behavioral results of our study showed poorer executive control skills in children of the youngest group (4–6 year olds) compared to older children and adults. Despite performing the experimental task at equivalent accuracy levels, the youngest group showed larger flanker interference score and larger impulsivity index than adults and older children (see **Figure 2**). Moreover, young children showed a significant smaller capacity to delay gratification compared to 7–10 and 10–13 year olds. All three measures suggest the existence of a major developmental change between preschool ages and middle-to-late childhood. This result is generally consistent with data from other developmental studies using a variety of tasks targeting executive functions, which also indicate that early to middle childhood constitutes an important developmental period of this function. This is for instance the case in studies using the dimensional card sorting task (Zelazo et al., 1996) inhibitory control (Bedard et al., 2002), and flanker tasks (Rueda et al., 2004a). Likewise, Wiersema et al. (2007) and Davies et al. (2004b) also found a decrease in the error vs. correct response time differences with age.

In addition to the behavioral level of analysis, we were able to study the neural basis of executive attention by registering electrophysiological patterns of activations during task performance. In our study, manipulation of the congruency of flankers modulated the amplitude of the target-locked N450 potential. This modulation was clearly observed in adults and 10–13 year olds in a group of frontally distributed channels (see **Figure 3**). In younger children, this modulation appeared to emerge later and to be sustained longer, although, as revealed by *t*-tests analyses, did not reach significance. Data in the literature about developmental changes in conflict-related modulations of target-evoked mid-frontal potentials greatly depend on the task being used. Several studies using Go–NoGo tasks have reported larger conflict effects in the N200/N450 amplitude by young children compared to older children and adults (Lamm et al., 2006; Hämmerer et al., 2010). This result suggests that the larger the effect on the amplitude of the N200/N450 the poorer the executive control efficiency. As a matter of fact, Lamm et al. (2006) reported an age-related decrease in N200/N450 amplitude between 7 and 16 years of age. However, using a flanker task with arrows, Ladouceur et al. (2007) found that only late adolescents (i.e., older than 14 years) and adults showed larger N200 amplitude in trials with incongruent flankers, while an early adolescents group also included in the study did not show the effect. Our results are consistent with data from this study as well as with those reported by Rueda et al. (2004b) where young children did not show negative amplitude modulations by flanker congruency but a sustained frontal effect after 500 ms post-target. Generally, the longer delay and duration of the effect in younger ages may, at least partially, explain young children’s poorer functional efficiency of the EAN.

Regarding neural processes of error monitoring, we found clear differences in the developmental trajectories of the ERN and P_e_ components. All age groups showed a clear P_e_ component. However, the ERN was not observed in 4–6 year old children. This result is consistent with prior data on the development of error processing during childhood (Davies et al., 2004b; Wiersema et al., 2007). There is evidence suggesting that the ERN consist of an early, and probably subconscious, signal of mismatch between the represented goal and the response being produced (Yeung et al., 2004). On the other hand, the P_e_ component appears to reflect accumulated evidence that an error was committed and the negative evaluation associated with it (Ridderinkhof et al., 2009; Steinhauser and Yeung, 2010). One possible interpretation is that the detection of errors in young children might depend to a greater extent on affective processes (an evaluation of the response and the negative outcome of this evaluation). Such processes would be slower than the subconscious mismatch thought to give rise to the ERN, and might involve the ventral (more affective) division of the ACC. In support of differential underlying mechanisms, some studies using dipole modeling have shown that the ERN and the P_e_ are generated in different brain regions (van Veen and Carter, 2002; Herrmann et al., 2004). Generally, both the dorsal and ventral vision of the ACC have been involved in executive control, the ventral division being particularly important in situations that are emotionally relevant (Bush et al., 2000). The ventral ACC facilitates executive control in situations signaled by emotion (Kanske and Kotz, 2011). Since each ACC division is associated with different cognitive mechanisms different developmental trajectories might be expected. Children in our study showed adult-like brain responses in the latency of the P_e_ component, a result that suggests that the ventral executive control system shows an earlier maturational trajectory than the cognitive dorsal system.

### CONFLICT AND ERROR PROCESSING AND SELF-REGULATION

The second goal of this study was to investigate the relation between the efficiency of EAN and the development of self-regulation. It has been suggested that mechanisms of executive attention are key to the development of self-regulatory skills (Rueda et al., 2011). Executive attention and the temperamental factor of EC are closely related concepts that depict different levels of analysis (i.e., cognitive and behavioral, respectively) of the ability to regulate behavior (Gerardi, 1997; Gonzalez et al., 2001; Simonds et al., 2007; Checa et al., 2008). Results of our study support the connection between cognitive measures of executive attention and the temperament factor of EC. Moreover, behavioral self-regulation measures and efficiency of EAN were related in our data. We found a correlation between higher impulsivity and poorer capacity to delay gratification and amplitude of the N450. As discussed above, larger N450 conflict effect is associated poorer executive attention efficiency. Thus, children showing poorer efficiency of the system at the neural level also show poorer regulatory skills at the behavioral level. Importantly, this result is obtained after age differences in the different measures are controlled for. These findings complement prior work supporting the existence of a link between efficiency of the EAN and individual differences in the ability to regulate actions (Posner and Rothbart, 1998; Rueda et al., 2011).

Previous research had linked impulsivity to difficulties in inhibitory control (Patterson and Newman, 1993; Barkley, 1997; Gonzalez et al., 2001; Enticott et al., 2006; Spinrad et al., 2012). Our data also reveal a positive correlation between the ability to inhibit inappropriate responses and impulsivity as well as a positive correlation between amplitude of the ERN and the ability to delay gratificaction. Previous studies have also shown a link between amplitude of the ERN and self- as well as social-regulation capacities (Santesso and Segalowitz, 2009). Moreover, individual differences in impulsivity were also associated with amplitude of the P_e_ component. These data are in line with previous studies showing that individuals who exhibited more impulsive behaviors displayed poor EAN efficiency, using the same or similar indexes of impulsivity as the one used in the present research (Pailing et al., 2002; Ruchsow et al., 2005), as well as using self-reported measures of impulsivity (Gonzalez et al., 2001; Heritage and Benning, 2013). In support of this relationship, there is evidence that children with ADHD, a disorder associated with impulsive behavior, show less efficiency in neural mechanism and structures within the EAN (Liotti et al., 2005; Wiersema et al., 2005; van Meel et al., 2007). All this evidence indicates that weaker and slower reactions related with conflict and error processing in frontal brain regions underlay behavioral patterns characterized by poor self-regulatory capacity. This conclusion is consistent with the role of the EAN in the Posner’s model (Posner et al., 2007).

According to Posner and Rothbart (2007), the EAN is involved in the regulation of emotional reactivity, both negative and positive. Our results are aligned with this idea. We found that higher flanker interference scores, indicative of poorer attentional control, were positively related to both negative affectivity as well as surgency. The use of attentional control to regulate emotions is thought to be supported by the system related to attentional selectivity (i.e., orienting network) in the early years, and relying on the developing EAN later on (Rothbart et al., 2011). The EAN is involved in controlling affect-related information through its connections with subcortical limbic structures such as the amygdala (Ochsner and Gross, 2004). Previous studies have associated dysfunctions of EAN with the inability to regulate emotions (Gehring et al., 2000; Ruchsow et al., 2005; Hajcak and Foti, 2008). Moreover, recent evidence shows that reduced activity in areas within EAN is associated with negative affect (Crocker et al., 2012).

Our data also revealed a negative correlation between surgency and the amplitude of the P_e_ potential. This suggests that excessive positive affect can impair some aspects of error processing. Several studies have reported that positive affect is associated with decreased planning abilities, task switching and worse inhibition abilities (Phillips et al., 2002; Mitchell and Phillips, 2007). We suggest that the development of the EAN, and subsequent enhanced attentional control, provides the attentional flexibility required to regulate approaching tendencies and resists temptations. This is particularly important when current conditions call for actions that conflict with future goals and those action are to be inhibited. The efficiency of EAN to control both positive/approaching as well as negative/avoiding tendencies is important for a broad range of aspects of children’s life such as morality and social adjustment (Kochanska et al., 2009), school readiness and academic performance (Checa et al., 2008; Checa and Rueda, 2011; Kim et al., 2013), and behavioral problems (Oldehinkel et al., 2004; Verstraeten et al., 2009).

The relation between efficiency of the EAN and regulation of approaching tendencies is not only restricted to reactive systems of temperament in our data. The ability to resist temptation in favor of long-term goals shows a positive relationship with amplitude of the ERN over and above age (see **Table 3**). There is evidence that success in the DoG task depends on the ability to regulate the attention during the waiting period (Mischel, 1974; Mischel et al., 1989). Additionally, imaging studies have shown that top-down control regions of the prefrontal cortex are activated during the delay period in DoG tasks (Casey et al., 2011; Heatherton and Wagner, 2011). Our data also show that children who were more able to delay gratification were the ones who better recruited the EAN during conflict resolution and error processing. Prior research has shown that performance of the DoG task in childhood predicts the efficiency with which the same individuals perform a Go/No-go task as adolescents and young adults (Eigsti et al., 2006).

## CONCLUSION

Data from this study inform about the development of diverse aspects of executive attention and self-regulation. Data from different domains (i.e., cognitive, temperament, and brain function) were taken into account. Results added to the evidence indicating that executive attention shows a period of major development during preschool years (Rueda et al., 2005b). By registering ERPs during performance of a flanker task, we were able to examine neural mechanisms related to conflict and error processing, and found that individual differences in efficiency of those mechanisms predict children’s ability to delay gratification and individual differences in impulsivity. Concretely, better error detection predicts larger percentage of delay choices and less impulsive behavior, whereas greater brain commitment (measured with amplitude of the N450 effect) in resolving conflict from incongruent flankers predicts smaller percentages of delay choices and more impulsive responses.

The scope of the current study was limited to the use of one particular experimental paradigm to explore brain mechanisms related to conflict processing and error detection. Flanker tasks are widely used in the literature to examine executive control, and the task utilized in our study had the advantage of adjusting the difficulty to the performance level of each participant; however, replicating the results of the study with other tasks (e.g., Stroop, Go–NoGo) would be desirable. Future studies might also benefit from using longitudinal designs in order to examine individual differences in the developmental trajectory of executive attention.

In sum, data from our study provide evidence that children showing a more efficient engagement of the EAN during development also show better self-regulation skills. In the recent past, mounting evidence is showing that neural mechanisms of executive attention can be enhanced by means of cognitive training (Rueda et al., 2005b). Interventions of this sort have the potential to also enhance children’s regulatory skills. For instance, we recently found that children trained in executive attention show better performance in a delay of gratification task compared to untrained peers (Rueda et al., 2012). Self-regulation is key to socialization and academic success (Rueda et al., 2010), therefore understanding the mechanisms underlying the development of this system as well as finding the best ways to boost its efficiency will be matters of great interest in future research.

## Conflict of Interest Statement

The authors declare that the research was conducted in the absence of any commercial or financial relationships that could be construed as a potential conflict of interest.
